# Association between route of illicit drug administration and hospitalizations for infective endocarditis

**DOI:** 10.1177/2050312117740987

**Published:** 2017-12-15

**Authors:** Olubunmi Olubamwo, Ifeoma N Onyeka, Alex Aregbesola, Kimmo Ronkainen, Jari Tiihonen, Jaana Föhr, Jussi Kauhanen

**Affiliations:** 1 Institute of Public Health and Clinical Nutrition, Faculty of Health Sciences, University of Eastern Finland, Kuopio, Finland; 2 Department of Forensic Psychiatry, Niuvanniemi Hospital, University of Eastern Finland, Kuopio, Finland; 3 Department of Clinical Neuroscience, Karolinska Institute, Stockholm, Sweden; 4 Helsinki Deaconess Institute, Helsinki, Finland

**Keywords:** Infective endocarditis, hospitalization, substance abuse, cohort study, injecting drug use, register linkage

## Abstract

**Objective:**

This study examined the association between the route of drug administration and being hospitalized for infective endocarditis among 4817 treatment-seeking illicit drug users in Finland.

**Methods:**

Cox regression models were used to examine the association between the route of drug administration and infective endocarditis hospitalization, adjusted for age, gender, and homelessness. Cases of infective endocarditis as a primary/main diagnosis were tracked using the 10th version of the International Classification of Disease code I33.

**Results:**

In all, 47 persons had a primary diagnosis of infective endocarditis. These 47 persons contributed a total of 95 hospitalizations and their total length of hospital stay was 1393 days. There was a statistically significant difference in hospitalizations between injectors and non-injectors (Log-Rank test p = 0.018). Univariate Cox model showed that injectors had higher hazard or risk for infective endocarditis hospitalization compared to non-injectors (hazard ratio: 2.04, 95% confidence interval: 1.12–3.73, p = 0.020). After adjusting for age, gender, and homelessness in the multivariate model, the elevated hazard among injectors compared to non-injectors remained statistically significant with adjusted hazard ratio of 2.12 (95% confidence interval: 1.11–4.07, p = 0.024).

**Conclusion:**

The study findings suggested a need to boost harm reduction measures targeting high-risk injecting and other health behaviors among injecting drug users in order to reduce their hospitalizations for infective endocarditis.

## Introduction

Illicit drug users are at risk of medical and infectious complications.^1^ One of them is infective endocarditis (IE) which is caused by infection and destruction of the smooth lining of the heart valves.^2^ Behaviors associated with drug use, for instance, drug injection, raise the susceptibility to IE among drug users.^3,4,5^ Predisposition to infection could also be due to other high-risk behaviors such as failing to clean skin and wash hands before injecting,^6^ increased frequency of drug injecting,^7^ and cleaning needles with saliva and dissolving drugs with saliva.^8^

IE is most commonly right-sided when associated with injecting drug use, and tricuspid valve is affected in 40%–69% of IE cases among drug injectors.^3^ Preponderance of tricuspid valve involvement among injectors has also been reported elsewhere.^9–11^ However, right- and left-sided infections could occur equally in injecting drug users.^12^ *Staphylococcus aureus* is the pathogen isolated in many cases of IE.^8,10,11,13^ However, other causative organisms include streptococcus,^10,14^ pseudomonas and klebsiella,^2^ candida,^15^ and aspergillus,^16^ or it could be polymicrobial involving combinations of several causative organisms.^8,10,17^

IE has been reported among patients with homelessness and substance use.^18^ IE varies with age, with young people being more affected than older adults, and IE tends to affect more males than females.^10,19^ However, a previous report suggests that there may not be any gender variation.^5^

Existing studies on IE among illicit drug users were conducted at international level and had limitations such as relatively short follow-up periods^6,7,9,13^ and being case reports.^14–17^ In Finland, IE hospitalizations among illicit drug users are less studied. Heiro et al.^20^ investigated a cohort of 303 patients with 326 hospitalizations for IE treated at Turku University Hospital during 1980–2004, and they found that intravenous drug use was one of the risk factors for recurrent IE hospitalizations. Other Finnish researchers^21^ conducted a trial involving patients followed up for 3 months after the first positive blood culture for *S. aureus*, and they found that IE was more common in *S. aureus* bacteremia in injecting drug users than non-drug users. However, the study populations for these previous researches were mixed and not exclusively drug-using populations. The primary objective of this study was to evaluate the association between the route of drug administration and being hospitalized for infective endocarditis in a cohort of treatment-seeking drug users in Finland. The secondary objective was to describe deaths from infective endocarditis during the same period.

## Methods

### Study design and population

This was a cohort of 4817 drug-using clients who sought drug treatment from the Helsinki Deaconess Institute (HDI) in Finland between 1997 and 2008. Of these 4817 clients, 3365 were men and 1452 were women, and their primary drugs of abuse at initial clinical consultation ranged from opiates, stimulants, cannabis, medication, alcohol, and other drugs. For a more detailed description of data collection, and the study population, see Onyeka et al.^22^ The clients’ data were linked to the Finnish National Hospital Discharge Register (FHDR) and the national cause of death register, and they were followed up to 31 December 2013 or death. The research ethics committees of the North-Savo Hospital District and the HDI, the Ministry of Social Affairs and Health of Finland, and appropriate municipal authorities gave approval for the study.

### Hospitalization and mortality data

Hospital discharge diagnoses and the causes of deaths were recorded using the 10th version of the International Classification of Disease (ICD-10) codes. This article considered only a subset of the clients whose main/primary hospital discharge diagnoses were recorded as ICD-10 code I33, denoting infective endocarditis (IE). Similarly, only clients whose underlying causes of deaths were recorded as ICD-10 code I33 were included in this article.

### Main outcome measure

To achieve the primary objective of this study, the main outcome measure was hospitalization for IE (hospitalized = 1, censored = 0), and the exposure variable was the route of administration of the primary drug (injecting = 1, non-injecting = 0). It is known that the association between injecting drug use and IE could be influenced by age and gender^8,10,19^ and homelessness.^18^

### Data analysis

Statistical Package for Social Sciences (SPSS) software was used for all analyses. Percentages and means were used to present the data. Kaplan–Meier survival analysis with Log-Rank test was used to compare survival for IE hospitalization between injectors and non-injectors. Cox regression analysis was used to examine the association between the route of drug administration and IE hospitalization. The multivariate models were sequentially adjusted for potential confounders using the following baseline variable: age (in years), gender (male = 1, female = 0), and homelessness (yes = 1, no = 0). Results were expressed as hazard ratios (HRs) and adjusted hazard ratios (aHRs) with 95% confidence intervals (CI). The p values ≤0.05 were considered statistically significant.

## Results

### Characteristics of the entire cohort

The majority of the clients (98%) were of Finnish nationality, 70% were males, and their mean age was 24.5 years (range: 11–65 years). They were mostly unmarried (92%) and 70% were from Helsinki municipality. Educational levels were low and three-quarters (75%) had elementary education. More than half (57%) were unemployed and nearly one-quarter (22%) were considered homeless. The primary drugs of abuse reported at baseline/initial clinical consultation included opiates (30%) mainly heroin and buprenorphine, stimulants (28%) mainly amphetamines, cannabis (19%), alcohol (21%), prescription medication (2%) mainly benzodiazepines, and other drugs (1%). Nearly half (45%) injected their primary drug. A more detailed description of the baseline characteristics of the cohort can be found elsewhere.^22^

### Infective endocarditis hospitalizations, deaths, and patterns of drug use

At the end of the follow-up in 2013, records from the hospital discharge register showed that 47 persons had main/primary diagnoses of IE. These people contributed a total of 95 hospitalizations, and their total length of hospital stay was 1393 days. During the same time period, six persons died from IE. Drug use information provided by the 47 clients during their initial clinical consultation is shown in Table 1. More than 4 in 10 persons (42.6%) reported stimulants as their primary drug of abuse, and the proportion of opiate users was also high at 29.8%. Almost two-thirds of the clients (62.2%) injected their primary drug and 37% injected regularly (i.e. ≥7 times/week) during the past month. Majority of them (93.6%) used more than one drug.

**Table 1.** Drug use characteristics (at initial clinical consultation) of the 47 clients hospitalized for infective endocarditis.

Characteristics	N = 47 clients
Primary drug of abuse	
Alcohol	8 (17.0%)
Cannabis	4 (8.5%)
Medication	1 (2.1%)
Opiates	14 (29.8%)
Stimulants	20 (42.6%)
Others	0 (0.0%)
Method of using primary drug	
Intravenous	28 (62.2%)
Smoking	4 (8.9%)
Oral	12 (26.7%)
Snorting	1 (2.2%)
Missing data	2 (–)
Past month use of primary drug	
No use	8 (17.4%)
≤once/week	7 (15.2)
2–6 times/week	14 (30.4%)
≥7 times/week	17 (37.0%)
Missing data	1 (–)
Number of drugs	
Single drug	3 (6.4%)
Multiple drugs	44 (93.6%)

### Association between injecting drug use and infective endocarditis

**Figure 1.**
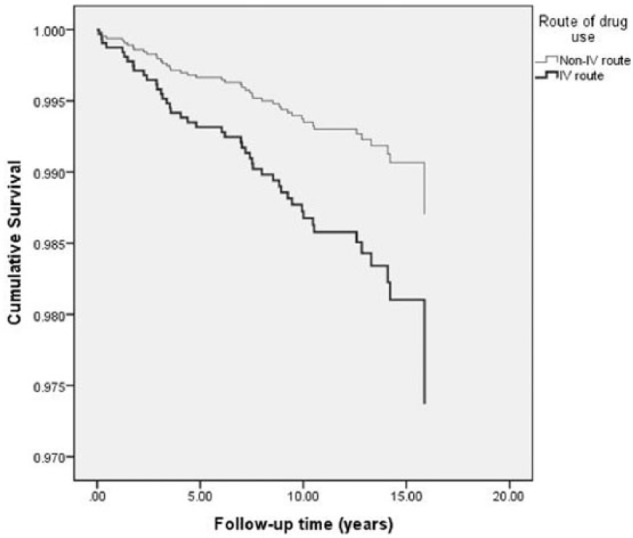
Cumulative survival of injectors and non-injectors during the follow-up period.

Kaplan–Meier analysis showed that there was a statistically significant difference in IE hospitalization between injectors and non-injectors (Log-Rank test *χ*²_[d = 1]_ = 5.6, p = 0.018). Univariate Cox model similarly showed that injectors had lower survival than non-injectors—the cumulative survival curves are presented in Figure 1. HRs of the association between route of drug administration and being hospitalized for IE are presented in Table 2. The univariate Cox model (i.e. model 1) showed that injectors had statistically significantly higher hazards for IE hospitalization compared to non-injectors (HR: 2.04, 95% CI: 1.12–3.73, p = 0.020). After adjusting for age, gender, and homelessness in the full multivariate model, the elevated hazard among injectors compared to non-injectors remained statistically significant at p = 0.024 (aHR: 2.12, 95% CI: 1.11–4.07).

**Table 2.** Association between injecting drug use and hospitalization for infective endocarditis, adjusted for potential confounders.

Baseline variables	Model 1 HR (95% CI)	Model 2 Adjusted HR (95% CI)	Model 3 Adjusted HR (95% CI)
Route of use, primary drug			
Injecting	2.04 (1.12–3.73)^‡^	2.13 (1.14–3.99)^‡^	2.12 (1.11–4.07)^‡^
Non-injecting	1.00 (ref)	1.00 (ref)	1.00 (ref)
Age (in years)		0.99 (0.95–1.03)	0.99 (0.94–1.03)
Gender			
Male		0.78 (0.42–1.44)	0.71 (0.38–1.33)
Female		1.00 (ref)	1.00 (ref)
Homelessness			
Yes			1.39 (0.71–2.72)
No			1.00 (ref)

Ref: reference; HR: hazard ratio; CI: confidence interval.

‡ Statistically significant at p ≤ 0.05.

## Discussion

In this study, we examined infective endocarditis (IE) hospitalizations in a cohort of 4817 Finnish drug users according to route of drug administration. We found that 47 persons had been hospitalized for IE at least once at the end of 2013, and they accumulated a total of 95 hospitalizations. The risk of hospitalization for IE in injectors was a 2.12 fold when compared to the non-injectors, after adjusting for age, gender, and homelessness. A total of six persons died from IE.

Our findings showed that nearly two-thirds of persons hospitalized for IE were injectors and this was consistent with previous international studies which reported high occurrence of IE among injectors.^7,9,13^ There was a statistically significant difference in hospitalization between injectors and non-injectors. Injection drug use can increase the risk of IE through a variety of mechanisms. Injected particulate matter may cause endothelial damage, with subsequent bacterial infection from high injected bacterial loads.^5,12^ The risk of IE in drug users could also be due to abnormalities in the immune function.^5,12^ The pathogens may have been introduced into the body from poor injection hygiene, injecting with unsterile equipment, and injecting contaminated drug solutions.^11,23^ High frequency of injecting drug provides more opportunities to introduce either pathogens or damage-causing particulate matter into the blood stream.^7,9^ Some of the substances injected (e.g. cocaine) can cause tissue and soft tissue damage, thereby elevating the risk of IE among injectors.^11^

Although studies in general population have documented an association between IE and age,^8,10,19^ this was not so in our study cohort of illicit drug users; age was not a significant determinant of IE. In the multivariate analyses, we found that gender was not an independent risk factor for IE. This finding was in contrast to previous reports of gender variation in IE.^8,10,19^ Moss and Munt^5^ similarly reported that both genders were equally affected in patients seen at their institution. In the same vein, homelessness did not seem to be independently associated with IE in this cohort. A possible explanation for this could be the small sample size of persons hospitalized for IE. Given that homeless people lack good hygiene, have high rate of injecting drug use, and might inject in unhygienic environment,^18^ research studies conducted with a larger sample size might be able to uncover an association.

In our study, only six deaths from IE were recorded at the end of 2013 and this was about the same with the number of deaths reported in an Italian cohort of drug users.^24^ There could be several explanations for this low number of deaths in our cohort. First, the publicly funded healthcare system in Finland ensures equal access to high-quality healthcare to residents of Finland. Second, nearly half of all clients in this cohort injected their primary drugs. Given this high level of injecting, it is likely that they had tricuspid valve IE which is more common in injecting drug users.^21^ Tricuspid IE is reported to have low mortality^9,20^ possibly because right-sided IE generally runs a benign course with relatively good prognosis^10^ but those with polymicrobial infection have high mortality.^8^

### Study limitation

Due to the register-based nature of this study, it was not possible to account for more detailed information from individual clients. Data were not available regarding the type of organisms that caused the IE, the valves that were affected, and whether it was a right-sided or left-sided IE. Neither was it possible to adjust for all the necessary risk factors such as previous history of valve disease and use of prosthetic valves and other devices. Despite these limitations, our study findings call for attention. There is a need to conduct more research on IE among drug-using populations possibly using larger samples and to formulate appropriate public health responses.

If possible, it might be interesting for future research using larger samples to assess the contributions of each individual type of illicit drugs to IE morbidity and mortality and the effect of substance abuse treatment on the risk of IE morbidity and mortality.

## Conclusion

In conclusion, IE was the primary hospital discharge diagnosis for 47 persons in this cohort of illicit drug users. The major predisposing factor for IE hospitalization was injection as a route of administration of the primary drug of abuse. Awareness of this may guide preventive strategies and increase recognition of IE in drug users. Addressing the underlying drug use problems, by giving support for drug cessation, should be given high priority. Other measures would include efforts to reduce high-risk injecting behaviors and other harm reduction measures, such as counseling about clean injection practices, and educating drug users about medical complications of drug use. In addition, counseling on maintenance of good overall personal hygiene, including oral hygiene and hand hygiene, can help reduce the risk of infections.
